# Predicting COVID-19 Vaccination Intention: The Determinants of Vaccine Hesitancy

**DOI:** 10.3390/vaccines9101161

**Published:** 2021-10-11

**Authors:** Nuno Fernandes, Daniela Costa, Diogo Costa, José Keating, Joana Arantes

**Affiliations:** 1 Psychology Research Center (CIPsi), School of Psychology, University of Minho, 4710-057 Braga, Portugal; id7743@alunos.uminho.pt (D.C.); keating@psi.uminho.pt (J.K.); joana.arantes@psi.uminho.pt (J.A.); 2 Department of Mechanical Engineering, University of Aveiro, 3810-193 Aveiro, Portugal; d.costa@ua.pt

**Keywords:** vaccine hesitancy, COVID-19, vaccination barriers, children vaccination, machine learning

## Abstract

Do people want to be vaccinated against COVID-19? Herd immunity is dependent on individuals’ willingness to be vaccinated since vaccination is not mandatory. Our main goal was to investigate people’s intention to be vaccinated and their intentions to vaccinate their children. Moreover, we were interested in understanding the role of the personal characteristics, psychological factors, and the lockdown context on that decision. Therefore, we conducted an online survey during the lockdown in Portugal (15 January 2021 until 14 March 2021). Participants completed a socio-demographic questionnaire, questions about their intentions of being vaccinated, concerns about the vaccine, a COVID-19 attitudes and beliefs scale, a COVID-19 vaccine attitudes and beliefs scale, and the Domain-Specific Risk-Taking (DOSPERT) Scale. Our results showed that from the 649 participants, 63% of the participants reported being very likely to have the vaccine, while 60% reported being very likely to vaccinate their children. We conducted two linear regression models, explaining 65% of the variance for personal vaccination and 56% of the variance for children vaccination. We found that the COVID-19 vaccine general beliefs and attitudes were the main determinants of vaccination intention. Additionally, our proposed artificial neural network model was able to predict with 85% accuracy vaccination intention. Thus, our results suggest that psychological factors are an essential determinant of vaccination intention. Thus, public policy decision makers may use these insights for predicting vaccine hesitancy and designing effective vaccination communication strategies.

## 1. Introduction

The COVID-19 pandemic has resulted in unprecedented social and economic disruptions, with a profound worldwide impact on public health, lifestyle, and food security [1]. Currently, more than 4 million people have died from COVID-19 worldwide, and more than 210 million people have been infected [2]. In Portugal, COVID-19 has killed more than 17 thousand people, and more than one million have been infected [2]. At the beginning of the year, Portugal implemented the second COVID-19 national lockdown to stop the spread of COVID-19 and to ease pressure on the National Health Service. Thus, the COVID-19 vaccine is a matter of public health since it is considered our main hope to tackle the coronavirus pandemic and to return to everyday life [3]. Across several countries, the national vaccination program has started, so finding the population’s intention of being vaccinated and knowing why some people do not intend to be vaccinated is important for improving the COVID-19 vaccination communication plan [4]. Additionally, we can predict vaccination intention at an individual and group level if we identify vaccine hesitancy determinants.

Herd immunity, estimated to occur when a large part of the population has been vaccinated and, hence, renders the coronavirus spread very unlikely, is dependent on the individuals’ willingness to be vaccinated [5]. Although the reported willingness to be vaccinated in Portugal (75%) was found to be higher than the average willingness in the European countries (73.9%), little is known about the actual numbers for the released vaccines since these judgments were performed based on a hypothetical vaccine [6]. With the vaccine available, it is essential to replicate these findings primarily due to the current national lockdown context that may influence people’s intention of being vaccinated. The proportion of people willing to receiving vaccination as soon as possible declined due to misinformation and the emerging concerns from governments across Europe [7]. The AstraZeneca vaccine has suffered constant setbacks due to issues related to blood clots [8], which decreased individuals’ trust in the vaccine and consequently reduced vaccination intention [9].

Vaccine hesitancy has been widely addressed, but few studies have evaluated the success of vaccination hesitancy reduction interventions [10]. Thus, there is a need to clarify the motives that influence vaccination intention in order to design effective intervention strategies. In a systematic review, there were several implemented strategies identified and grouped into the following categories: individual/social group influences; vaccine and vaccination-specific issues; and contextual issues [10]. The authors found that the most effective interventions employed multiple strategies, such as increasing vaccination knowledge and awareness, improving convenience and access to vaccination, and engaging influential leaders to promote vaccination.

Specifically, COVID-19 vaccine hesitancy should be strongly considered since the vaccine’s safety and effectiveness have been highly discussed in social media due to the record time of its development process and its unknown long-term side effects [11]. The literature suggests that individual characteristics play a significant role in the acceptance of the COVID-19 vaccine: older age [12,13], male gender [12,14], or religion [14] were frequently reported as good predictors of vaccination intention. 

Additionally, the fear of facing adverse side effects from the vaccine may prevent the achievement of a global immunity as people commonly intend to avoid losses more than they avoid gains. This concept was defined by Kahneman and Tversky [14] as loss aversion. The concept has already been used to frame vaccination intention as a trade-off between risks [15]. However, there is still a mix in literature as to whether higher risk-averse people are more afraid of the potential vaccine side effects or, on the other hand, whether they show stronger intentions of being vaccinated to avoid the negative consequences of the actual disease [4,16]. Despite the fact that the role of emotion in COVID-19 vaccine communication has already been discussed, with fear being associated with vaccine hesitancy [4], little is known about a possible loss aversion effect. 

Framing the COVID-19 vaccine communication plan regarding the vaccination intention’s psychological determinants may significantly diminish vaccine hesitancy [17]. For example, the theory of planned behavior (TPB) predicts that social norms may influence vaccination intention by shaping the individuals’ perception about the socially desired behavior—injunctive norms [18]. Social norms are values and beliefs shared within a population, guiding the group’s behavior without explicit laws [19]. Thus, it is essential to identify the shared beliefs and attitudes towards the coronavirus and the vaccine. 

Furthermore, contextual issues such as culture, politics, media environment, or influential leaders should also be considered when addressing communication strategies as they are systematically reported as determinants of vaccine hesitancy [10]. The increase in COVID-19 new cases in several countries lead governments to declare national lockdowns. Under current restrictions, people must stay at home, with limited exceptions for permitted reasons. Therefore, the actual conditions are of great importance to observe whether adverse settings in which the coronavirus’s negative impacts are highlighted (e.g., staying at home) influence vaccine hesitancy. 

During the lockdown, individuals may recall coronavirus’s negative consequences easier and, consequently, heavily weigh its potential effects. One possible explanation, supported by the availability heuristic [20], is that people perform likelihood judgments, e.g., calculate the risk of becoming seriously ill, based on the ease of retrieval of certain events. In the case of the COVID-19 pandemic, they may perform judgments based on the number of daily new positive cases or for how long they have been in lockdown. 

## 2. Parental Vaccination Intention

Despite the efforts placed towards the rapid development of a COVID-19 vaccine, the actual clinical trials are not clear about the desired dose or the possible side effects for children (under 18 years old) [21]. Children can also become infected and transmit, and develop clinical complications from the coronavirus, so there is a need to approve children’s COVID-19 vaccine uptake [21]. However, the success of vaccinating this age group relies on the parents’ willingness to vaccinate their child/children. Parental vaccine hesitancy has also been frequently explained by psychological factors such as a higher perceived risk of the child experiencing heavier adverse effects from the vaccine than from the actual disease or the perceived ability to control children’s exposure to the disease [22]. 

Relative to the COVID-19 vaccine, parents reported to be more likely to vaccinate themselves than their child/children, and the main reasons for not accepting a children’s vaccine were as follows: not enough evidence; safety concerns; and the belief that children are hardly affected [23]. Given the delay in COVID-19 vaccine clinical trials for children, they will be the last age group to receive the vaccine [21]. Thus, it becomes essential to explore the most critical factors for explaining parental vaccine hesitancy to prevent widespread parental refusal.

This study answers a public health issue and the achievement of group immunity. Our main objective is to find people’s willingness to be vaccinated and to vaccinate their children. Then, we aim to clarify the underlying factors of that choice and uncover why people do not intend to be vaccinated. These motives will be framed under personal characteristics, psychological factors, and context. Following previous research [6,13], which found that psychological factors were the main predictors of vaccination intention, our first hypothesis is that the beliefs and attitudes towards the COVID-19 vaccine will be the main determinants of vaccination intention. Thus, we expect that individuals with more positive beliefs and attitudes about the COVID-19 vaccine will show higher vaccination intentions. 

Following Tversky’s and Kahneman’s [20] availability heuristic predictions, we expect that as the number of days in lockdown increases, people will be more prone to being vaccinated in order to mitigate the adverse effects of the pandemic. Relative to personal characteristics and similarly to previous studies [12,14], we expect that age will positively affect vaccination intention. Other socio-demographic variables will also be included in further exploratory data analysis. Ultimately, we intend to build an artificial intelligence model for predicting vaccination intention. We hope that our findings can provide public policy managers with powerful insights into predicting and avoiding widespread COVID-19 vaccine hesitancy.

## 3. Method

### 3.1. Participants

Our sample consisted of 649 participants of the general adult population living in Portugal, ranging from 18 to 84 years old (*M* = 30.71; *SD* = 12.48). The sample was comprised 495 females (76%), 152 males (23%), and two who did not disclose their gender (0.3%). A total of 113 participants (17.4%) reported having minor children. The exclusion criteria were as follows: (1) have already been vaccinated against the COVID-19 and (2) not living in Portugal. All participants provided informed consent according to the Helsinki Declaration. None of the participants received any monetary compensation, and they were recruited through institutional email and online social networks (e.g., Facebook).

### 3.2. Materials

**Socio-demographic variables questionnaire** (**Q-SV**)**:** Participants were asked about their gender, age, nationality, religion, highest education qualification, area of residence, socioeconomic status, and their actual professional situation. Moreover, we asked participants if they were in a romantic relationship and if they had children. Detailed information is provided in Appendix A (Table A1).

**Questionnaire outcome measure.** The intention to be vaccinated was measured by asking participants, “*what is your intention to be vaccinated?”* [13]. Moreover, we measured participants’ intention to vaccinate the children. For parents of children under 18 years old, we asked them, “*what is your intention to vaccinate your child/children, when possible?*”. Otherwise, we asked participants to “*imagine you have one or more minor children. What is your intention to vaccinate your child/children, when possible?*”. These two questions, relative to the intention of vaccinating themselves and children, were presented in random order and answered on a 7-point scale, from “*none*” to “*high*”.

**Questionnaire regarding lockdown impact.** We were concerned about the current lockdown impact on people’s willingness to be vaccinated. Therefore, we registered the day people answered the questionnaire to establish how long they have been in lockdown. Participants were asked to rate four questions regarding the lockdown period: (a) how much their lives have changed due to the lockdown period on a 7-point Likert scale, from 1, *“Very little or nothing,”* to 7, “*Extremely*”; (b) with whom they were living during this period; (c) the current professional situation (if working/studying from home or working/studying outside the home); and (d) if their economic condition has changed due to the current lockdown.

**COVID-19 attitudes and beliefs** [13]. Participants were presented with a series of eight statements concerning their attitudes and beliefs about COVID-19 [13]. They were asked to rate these questions on a 7-point scale ranging from “strongly disagree” to “strongly agree.” The scale was translated to Portuguese by using a forward-backward method. Supported by previous findings in the UK [13], we asked participants two additional questions about the risk of the COVID-19 to themselves and other people in Portugal on a 5-point scale from “*none*” to “*high*” and if they thought they “h*ave had, or currently have, COVID-19.*” Participants who answered “*I have definitely had it or definitely have it now*” or “*I have probably had it or probably have it now*” were classified as having had coronavirus, while participants who reported “*I have probably not had it and probably don’t have it now*” or “*I have definitely not had it and definitely don’t have it now*” were classified as not having had coronavirus. 

**COVID-19 vaccine attitudes and beliefs** [13]. The attitudes and beliefs about the COVID-19 vaccine were measured using Sherman’s et al. [13] proposed scale constituted of 20 statements. This scale was also translated into Portuguese by using a forward-backward method. An illustrative item is “a coronavirus vaccination will be too new for me to be confident about getting vaccinated.” Participants were asked to rate the items on a 7-point scale ranging from “strongly disagree” to “strongly agree”. Furthermore, we asked participants an additional question about their concerns regarding the COVID-19 vaccine. Participants selected, between a set of options, their concerns towards the vaccine, for example, “the vaccine was developed in record time,” “I belong to a group risk,” or “the vaccine could contain a microchip.” These options were formulated based both on public discussions on social media and literature regarding vaccine hesitancy [10,11,13].

**Domain-specific risk taking** (DOSPERT [24]; Portuguese Version [25]). This instrument tests risk aversion and was measured using Portuguese translation of the DOSPERT [25], which is a revised and shorter version (with 30 items) of the original scale [24]. The DOSPERT’s risk-taking sub-scale is applied by asking participants to “please indicate the likelihood that you would engage in the described activity or behavior if you were to find yourself in that situation.” For each of the 30 items, they are grouped among five different dimensions: ethical; economic; health/safety; recreational; and social on a 7-point scale ranging from “extremely unlikely” to “extremely likely. 

### 3.3. Ethics

This study received ethical approval from the Ethics Committee for Research in Social and Human Sciences of the University of Minho (reference: CEICSH 015/2021).

### 3.4. Procedure

The online questionnaire was developed in Qualtrics software (Qualtrics, Provo, UT, USA) [26]. The questionnaire was available for the entire lockdown period (from the 15 January 2021 to the 14 March 2021). Participants completed a socio-demographic questionnaire followed by questions about the outcome measures and questions about the lockdown impact. To finalize, participants filled three questionnaires presented in random order: a COVID-19 attitudes and beliefs scale, a COVID-19 vaccine attitudes and beliefs scale, and the Domain-Specific Risk-Taking (DOSPERT) Scale. The participants took, on average, 10 min to complete the survey.

## 4. Data Analysis

Statistical analyses were performed using R [27] in RStudio Version 1.4.1103 (R Foundation for Statistical Computing, Vienna, Austria) [28], while we used the scikit-learn library [29] from Python 3.9.2 for the machine learning model. To explain the motives behind the intention to have/not to have a COVID-19 vaccine, we built two multiple linear regression models (MLR): one having the participants’ intention to vaccinate themselves as the outcome variable, and the other having the intention to vaccinate children as the outcome variable. Our objective was to find the two most parsimonious models. Thus, we constructed two nested models, with only the significant predictors found in the first exploratory models constituting all the measured variables from personal characteristics, psychological factors, and context. Ordinal and multinomial predictors were converted into dummy variables. The adjusted R-squared values obtained justify the total explained variance in the two regression models. Additionally, we trained a machine-learning algorithm to forecast people’s willingness to be vaccinated against COVID-19. We used an artificial neural network (ANN) model for which its inputs were selected based on the results of the MLR models.

## 5. Results

### 5.1. Self and Children Vaccination Intention

**Figure 1:**
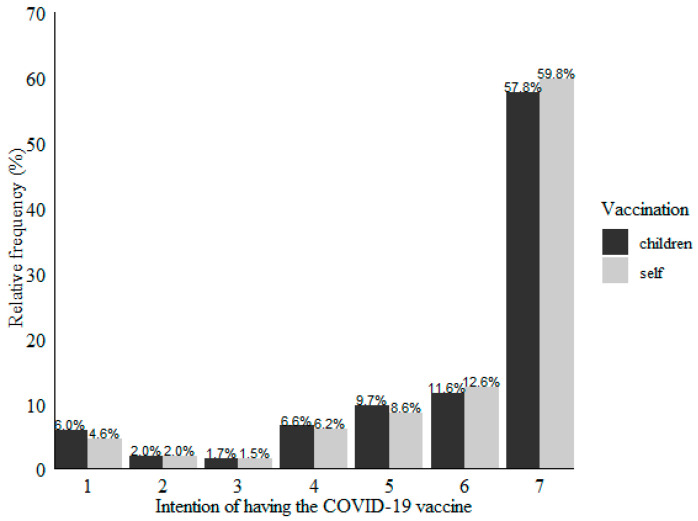
Self and children vaccination intention (1 = “extremely unlikely” to 7 = “extremely likely”).

Participants’ vaccination intention is expressed in Figure 1. Vaccination intention of the participants showed a left-skewed distribution (*M* = 6.03; *SD* = 1.63; *ME* = 7.00). Vaccination intention of the participants’ children also showed a left-skewed distribution (*M* = 5.91; *SD* = 1.74; *ME* = 7.00). Participants’ intention of receiving the COVID-19 vaccine was, on average, higher than the intention of vaccinating their children, *t*(648) = 3.14 and *p* = 0.002.

### 5.2. Vaccination Concerns

**Figure 2:**
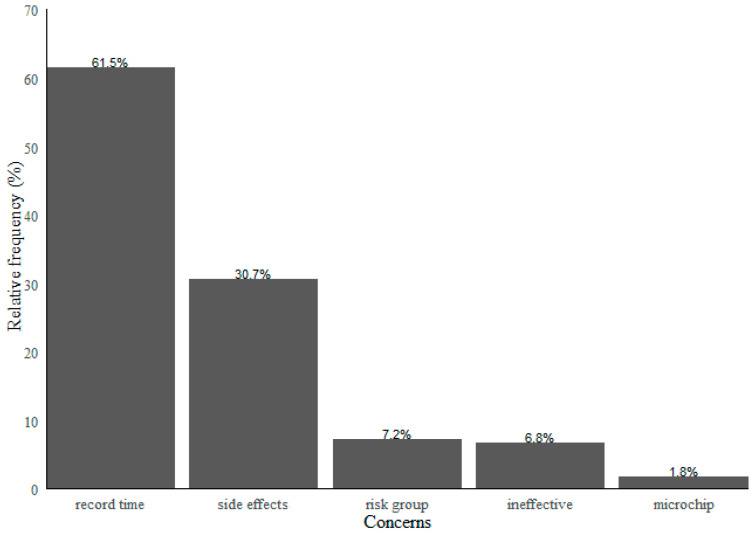
Major reported concerns relative to the COVID-19 vaccine.

Participants indicated that their primary concern of receiving the COVID-19 vaccine was the record time of its development process followed by the possible adverse side effects of the vaccine. Moreover, the participants frequently reported belonging to a risk group and doubting the vaccine’s effectiveness as their primary concerns. These results are presented in Figure 2. 

### 5.3. Dimensionality Reduction from the COVID-19 Attitudes and Beliefs Scale

We performed two principal component analysis (PCA), one for the COVID-19 attitudes and beliefs scale and the other for the attitudes and beliefs about the COVID-19 vaccine scale. Relative to the coronavirus scale, three main dimensions were extracted: the perceived threat of COVID-19 (*α* = 0.58), trust in the management of the COVID-19 (*α* = 0.84), and the impact of COVID-19. Thus, confirmatory factor analysis was performed, which showed a comparative fit index of (CFI) = 0.94, a Tucker–Lewis index of (TLI) = 0.90, and a root mean square error of approximation (RMSEA) = 0.07. Data are presented in Appendix B (Table A2).

### 5.4. Dimensionality Reduction from the COVID-19 Vaccine Attitudes and Beliefs Scale

From the vaccine scale, four main dimensions were extracted: general COVID-19 vaccination beliefs and attitudes (including, e.g., subjective norms, behavioral control, anticipated regret, and vaccine adverse side effects); the “others” intention of being vaccinated; the perceived knowledge sufficiency; and the return to normal life. Thus, a confirmatory factor analysis was performed which showed CFI = 0.82, TLI = 0.79, and RMSEA = 0.09. Data are shown in Appendix C (Table A3).

### 5.5. Multiple Regression Models of Self and Children Vaccination Intention

We built two nested multiple linear regression models, one for explaining self-vaccination intention and the other for explaining the intention to vaccinate children consisting of only the predictors that showed a significant effect in the complete original models (with all the measured variables). 

The first model, relative to the self-vaccination intention, explained 65% of the variance (*F*(3, 645) = 397.6 and *p* < 0.001). It was observed that positive beliefs and attitudes towards the vaccine significantly predicted self-vaccination intention (*β* = 0.75, *t*(645) = 31.38, and *p* < 0.001), as did the perceived risk of the COVID-19 (*β* = 0.07, *t*(645) = 3.03, and *p* = 0.003), while the perceived knowledge sufficiency relative to the coronavirus and the vaccine negatively predicted self-vaccination intention (*β* = −0.05, *t*(645) = −2.05, and *p* = 0.040).

The model with the intention to vaccinate the children entered as the outcome variable explained 56% of the variance (*F*(3, 645) = 278.9; *p* < 0.001.) It was observed that positive beliefs and attitudes towards the vaccine significantly predicted the intention to vaccinate children (*β* = 0.72; *t*(645) = 23.47; *p* < 0.001), as did COVID-19 perceived threat (*β* = 0.09; *t*(645) = 3.21; *p* = 0.001). On the other hand, perceived knowledge of the coronavirus and the vaccine, despite the fact that it approached the significance level in the global model, did not show a significant effect in this most parsimonious model (*β* = −0.04; *t*(645) = −1.53; *p* = 0.13). 

### 5.6. The Predictive Power of the General Beliefs and Attitudes towards the Vaccine

**Figure 3:**
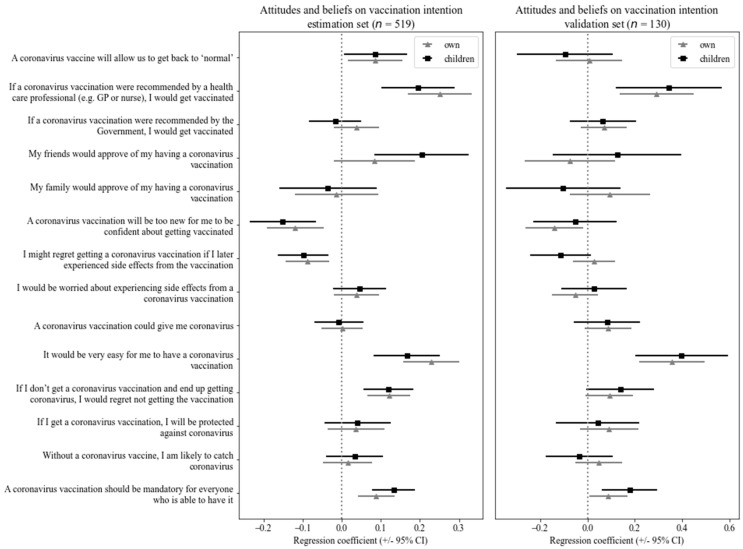
Results of multiple linear regression for predicting vaccination intention from general attitudes and beliefs towards the vaccine.

To understand the contribution of each item from the general attitudes and beliefs towards the vaccine component for predicting vaccination intention, we performed two multiple regression models: one for explaining self-vaccination intention (*F*(14, 634) = 101.9; *p* < 0.001; $R^{2}$ = 0.69); and the other for explaining children vaccination intention (*F*(14, 634) = 69.44; *p* < 0.001; $R^{2}$ = 0.61). We would like to highlight that health care professionals’ recommendation on receiving the vaccine had a significant positive effect for both self-vaccination intention (*β* = 0.25; *t*(634) = 6.97; *p* < 0.001) and for children vaccination (*β* = 0.22; *t*(634) = 5.09; *p* < 0.001), while the lack of confidence because the vaccine was too new showed a negative effect both for self-vaccination intention (*β* = −0.13; *t*(634) = −3.95; *p* < 0.001) and for (*β* = −0.13; *t*(634) = −3.31; *p* = 0.001). Then, in order to ensure reproducibility of our results, we performed the same analysis in a subset comprising 80% of participants and repeated it in the remaining 20%. The regression coefficients for each item are presented in Figure 3 (code retrieved from [30]).

### 5.7. Machine Learning (ML)

We trained an artificial neural network (ANN) machine learning model to predict individuals’ willingness to be vaccinated with respect to the recently released COVID-19 vaccine. ANNs were inspired by how the human brain works and are vastly used for pattern recognition and classification problems [31]. ANNs have proven useful, for example, for predicting the COVID-19 outbreak [32,33].

To develop our ANN, the entire dataset (649 participants) was divided into two parts in a ratio of 80/20: a training set consisted of 519 answers where the model learned and adjusted its predictions, and a testing set consisted of 130 entries in which we tested the quality of the model’s predictions. During the training phase, hyperparameter tuning was achieved by using exhaustive search through GridSearch with five parameters to find the model with the highest 3-fold cross-validation accuracy. The best model showed an 80% cross-validation accuracy and was defined by the following: a ReLu (rectified linear unit) activation function; a hidden layer with three nodes; a learning rate of 0.0001; the Adam (adaptative moment estimation) optimizer algorithm; and 1000 maximum iterations. 

**Figure A1:**
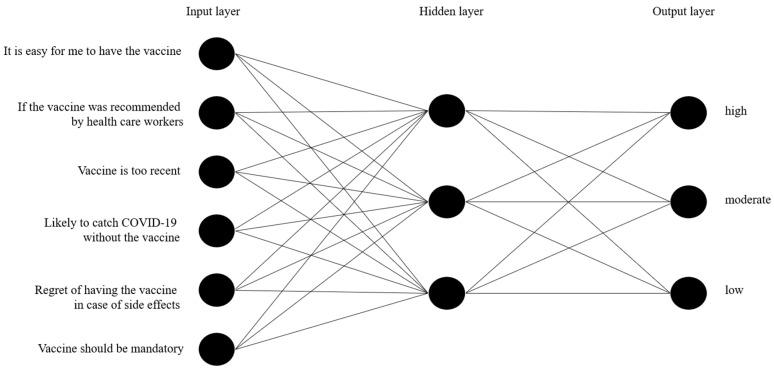
Multi-class artificial neural network (ANN) for predicting COVID-19 vaccination intention.

The ANNs have three layers: an input layer, hidden layers, and an output layer (a global view of our model is presented in Appendix D and is available as (Figure A1). The output layer consisted of a multi-classification problem in which the model was trained to predict whether the person would have the following: have the COVID-19 vaccine for sure; have moderate intention; or have low intention. These three categories were created from the 7-point Likert type question “what is your intention to be vaccinated?” where “7” was coded as having the vaccine for sure, “6–4” as having moderate intention, and “3–1” as having a low intention. These three categories were formulated from a theoretical and empirical perspective. Theoretically, we were interested in finding whether the person would have any doubts about having the vaccine, so any answer below “7” was considered showing a certain degree of vaccine hesitancy. Empirically, as the responses followed a left-skewed distribution, with more than half participants answering “7” (62.6%), it made sense to try to identify, in the general population, people who have some sort of vaccine hesitancy and to what degree that hesitancy manifests (high vs. moderate hesitancy). 

**Figure A2:**
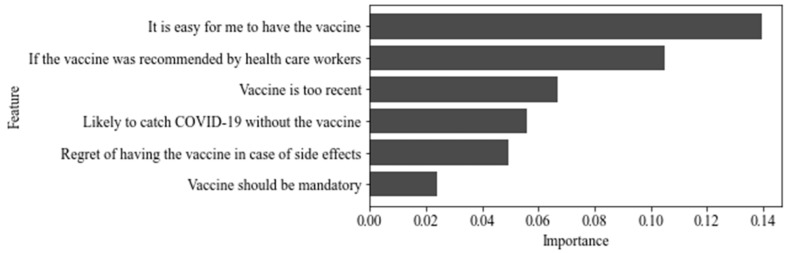
Relative feature importance of the ANN model.

The input features were chosen based on previously identified critical components through a multiple regression model: general attitudes and beliefs towards the vaccine, COVID-19 perceived risk, and perceived knowledge. Initially, we tested the model with 17 items relative to those three components. Then, we computed the relative importance of features and selected those that contributed the most to the model’s performance. The final model comprised six inputs, all belonging to the previously extracted component of general attitudes and beliefs towards the vaccine. The questionnaire’s items were used as inputs and not the components extracted from the previous PCA analysis since the model showed a higher performance with the items entered as features. The relative importance of each feature is presented in Appendix E (Figure A2). 

In the testing phase, the model showed an accuracy rate of 85% for predicting participants’ vaccination intention, with a sensitivity of 100% for identifying people with high vaccine hesitancy. When compared with other machine learning types of algorithms, our ANN showed the highest accuracy in a 10-fold cross-validation test. The confusion matrices for the model’s predictions vs. the actual values in the testing set, as well as additional evaluation metrics (precision, recall, and the F1-score) for the adequacy of the model to each class, are presented in Appendix F (Table A4 and Table A5). 

## 6. Discussion

The main aim of the present study was to find people’s intention of being vaccinated against COVID-19 in Portugal and to vaccinate their children. Moreover, we expected to find the underlying factors behind that decision and the genuine concerns towards the vaccine. For this reason, the survey analyzes participant’s characteristics, psychological factors, contextual factors, and concerns about the vaccine. Ultimately, our goal was to predict vaccination intention by using a machine learning model with the determinants of vaccine hesitancy as inputs. Therefore, we first hypothesized that psychological factors would be the significant determinants of vaccination intention, especially the general beliefs and attitudes towards the vaccine. Moreover, we expected that the lockdown would positively affect individuals’ willingness to be vaccinated and that age would positively predict vaccination intention.

Most people reported having full intentions of being vaccinated and vaccinating their children. Although nearly half the participants were not sure about having the vaccine, the principal reported concerns were included the record time of its development process and its possible adverse side effects. 

In order to understand the determinants of vaccine hesitancy, we constructed two multiple regression models for explaining self and children’s vaccination intention. We found that all significant predictors belonged to the group of psychological factors, with positive beliefs and attitudes towards the vaccine being the significant determinants of vaccination willingness. However, we cannot conclude that neither contextual factors, such as the number of days in lockdown, nor personal characteristics such as age influenced vaccination intention. Regarding self-vaccination intention, we also observed that the perceived risk of contracting the coronavirus was positively linked to a higher vaccination intention. In contrast, the perceived knowledge about the disease and the vaccine seemed to reduce vaccination intention. Relative to the intention of vaccinating the children, the model showed that a higher perceived COVID-19 threat was positively linked with the willingness to vaccinate the children.

Finally, our neural network machine learning model for predicting vaccination intention could be operationalized by providing the model with inputs relative to six questions from the COVID-19 vaccine attitudes and beliefs scale [13]. The model was able to predict with 85% accuracy whether the individual would have low, moderate, or high intention of being vaccinated against COVID-19. 

### 6.1. Theoretical Implications

This study presents essential findings for understanding COVID-19 vaccination intention, its determinants, and the main concerns about the vaccine. Our results suggest that nearly half the Portuguese population shows a certain degree of vaccine hesitancy. Despite vaccine hesitancy being not as high as other authors previously found for the same population [34], the results that show 56% of individuals reporting that they preferred delaying vaccine uptake and 9% reporting they refused are still alarming. The most-reported concerns are related to the vaccine’s side effects [6,13] and lack of trust in the vaccine’s development process due to its record time [11]. Moreover, despite the priority in vaccinating particularly vulnerable populations, such as cancer patients [35], belonging to a risk group was commonly reported as a concern about having the vaccine by the participants in our study. This is explained since cancer patients’ leading concern is the vaccine’s possible side effects, similarly to the general population [36].

Vaccine’s general beliefs and attitudes, containing questions regarding the vaccine’s side effects, family and “friends” approval, and the recommendation by government and public health professionals, among others, were found to be the most determinant factors in predicting COVID-19 vaccination intention. These findings support existing literature concerning the effect of social norms on COVID-19 vaccination intention [37,38]. Moreover, COVID-19 perceived risk and perceived threat and the perceived knowledge about the coronavirus and the vaccine were also significant predictors for explaining self-vaccination intention and children’s vaccination intention, which is particularly relevant as these predictors all belong to the group of psychological factors, similarly to previous studies [6,13]. Surprisingly, individuals with higher perceived knowledge about the coronavirus and the vaccine showed lower vaccination intentions. In an experimental study, it was observed that exposure to misinformation decreased participants’ willingness to be vaccinated, especially for scientific-sounding misinformation [7]. Linden et al. [39] also warned of the adverse impact of misinformation on strategies to tackle the pandemic since COVID-19 misinformation had been widely spread on social media. Thus, the negative effect of perceived knowledge on vaccination intention could be explained by participants’ prior exposure to misinformation relative to either the disease or the vaccine.

Since only psychological factors were determinants of vaccination intention, we cannot support previous findings of the importance of personal characteristics. A possible explanation for the absence of significant effects is that the influence of personal characteristics on vaccination intention varies between populations. In France [12] and UK [13] age had a positive effect on vaccination intention, while in the US [14] it had no effect. Moreover, men in the US [14] and in France [12] showed higher vaccination intention than women, while this effect was not observed in the UK [13]. Finally, religiosity decreased vaccination intention in the US [14] but showed no effect neither in France [12] nor UK [13].

Additionally, contrary to our expectations, the number of days in lockdown did not influence vaccination intention. Fridman et al. [40] tested, by using a longitudinal study, whether the perceived COVID-19 threat would increase over time and, consequently, increase the positive attitudes towards the vaccine and the vaccination intention but found an overall decrease in vaccination intention. Thus, similarly to previous findings [40], we cannot conclude that changes in context affect people’s willingness to be vaccinated [10]. Despite the lockdown’s restrictions on individuals’ lives, with the decreasing number of COVID-19 infections during the lockdown [41], people could have believed that vaccination would no longer be necessary. Finally, despite our findings of some of the psychological factors behind vaccination intention, others remain to be clarified, as with risk aversion. Risk aversion did not significantly affect our multiple regression models, and it decreased the model’s performance when entered the ML model. In this manenr, we cannot provide further insights into the debate on whether risk aversion has a positive or negative role in vaccine hesitancy [42,43].

With respect to children’s vaccination, our results support previous findings of the parents’ higher intentions of receiving vaccination against COVID-19 themselves compared to the intention of vaccinating their children [23]. One possible explanation is that the COVID-19 perceived threat for children is so low that the advantages of receiving the vaccine are inferior to the vaccine’s potential adverse side effects [23]. Our results support this influence of the COVID-19 perceived threat on child vaccination, as higher degrees of COVID-19 perceived threat predicted increased willingness to vaccinate children.

The ANN model supports the results of the multiple regression models, with items regarding the general beliefs and attitudes towards the vaccine being the most relevant features for predicting vaccination intention. This study shows that an algorithm can predict vaccination intention of a given population with good accuracy even with only a few inputs. In sum, ANNs complement linear regression models by capturing non-linear relationships and predicting subsequent behavior [44]. ML models have proved to be important for understanding and predicting the coronavirus spread [45], and our results suggest that they can also be applied to the vaccine hesitancy issue.

### 6.2. Practical Implications

Now that COVID-19 vaccines are available and the vaccination plan is being carried out, addressing vaccination intentions is of great importance in order to prevent widespread vaccine hesitancy. Our findings suggest that vaccine hesitancy should be cautiously considered. Although most participants reported wanting to be vaccinated and wanting to vaccinate their children, a large proportion was doubtful. Interestingly, participants who reported the maximum intention of being vaccinated still shared some of the concerns relative to the vaccine. Thus, communication strategies should consider people’s lack of trust in the vaccine’s development process and safety. 

Public policymakers may find our results about the determinants of the vaccination intention essential for designing effective strategies for reducing vaccine hesitancy. The most significant predictor was the general beliefs and attitudes towards the vaccine, which encompasses, among other attributes, the vaccine’s side effects, others’ approval, and expert recommendation. Thus, communication plans may use people’s general beliefs and attitudes about the vaccine to reinforce the driving forces and reduce the restraining forces of the vaccination behavior [46]. For example, similarly to previous findings about the human papillomavirus vaccination [47], framing COVID-19 vaccination messages using injunctive norms could increase the interest in seeking additional information about the vaccine and, consequently, enhance confidence in receiving vaccinations. Additionally, our findings suggest using an authority principle of persuasion [48] may reduce vaccine hesitancy. Therefore, experts (health professionals) and authority figures (government’s members) should communicate the vaccine’s importance and safety.

Additionally, our findings suggest that parental hesitancy towards expected child vaccination against COVID-19 should also be considered, as participants reported a lower intention of vaccinating their children than compared to vaccinating themselves. In addition to the already discussed strategies, we would like to highlight the importance of raising awareness about the actual COVID-19 threat for children, especially with the new variants of the virus [49], as this was found to be a determinant of parents’ intention to vaccinate their children.

The proposed ANN may contribute to managing the vaccination plan since it allows us to predict people’s intention of receiving vaccinations against COVID-19. The model’s simplicity is another practical advantage since it only has six inputs and performs relatively well. Thus, with the results of these six questions, we can predict vaccination intention without having to ask people about their vaccination intentions directly.

### 6.3. Limitations and Further Studies

This study presents some limitations that should be considered. The main limitation concerns our sample characterization. The number of female participants vastly exceeded the number of male participants. The participants were, on average, relatively young, and only a few participants reported having primary and basic education as their maximum qualification. Thus, future studies must not neglect the commonly reported effects of gender, age, and education on COVID-19 vaccination intention [12,13,14].

Our machine model approach was not extended to predict children’s vaccination since only 113 participants reported having minor children. However, future studies with a larger sample size may apply our ANN for predicting parents’ intention of vaccinating their children now that child vaccination has been approved.

Additionally, our ML model was trained and validated with data from the Portuguese population. Generalization of the proposed model to other populations should be taken cautiously since predictors of vaccination intention may differ due to societal and cultural beliefs [12,13,14]. Therefore, we highlight the importance of future studies validating our model for different populations. Finally, our sample size comprised 649 participants with unleveled distributed classes (a low percentage of people showing low intentions of receiving vaccination), which poses a risk of overfitting and, consequently, results in lack of generalization power [50]. Global datasets could be a solution to this problem since the code is freely available online. However, a global approach to solve COVID-19 related issues poses limitations since data variability may reduce the models’ performance [51]. Therefore, a trade-off between performance and generalization power should be considered when applying the proposed ANN to other populations.

## 7. Conclusions

The present work addresses COVID-19 vaccination intention. We examined people’s willingness to be vaccinated and the underlying factors behind that decision. We found that 63% of the participants were certain about receiving vaccinations, and 60% were sure about vaccinating their children. The determinants of vaccination intention were attributed to psychological factors, with general beliefs and attitudes towards the vaccine being the most important predictor. Furthermore, our findings suggest that the significant concerns about having the COVID-19 vaccine were due to the record time of its development process and its possible side effects. Therefore, strategies that aim to fight vaccine hesitancy should consider these determinants and concerns in their communication campaigns, which may be complemented by using our proposed algorithm to predict people with strong vaccine hesitancy. All in all, our research provides important insights to help the fight against the vaccine hesitancy problem.

## Data Availability

Data is available at https://doi.org/10.17605/OSF.IO/TR2P3. Analysis code was written in R and Python and is available at https://nunokf.github.io/Predicting-COVID-19-Vaccination-Intention-The-Determinants-of-Vaccine-Hesitancy-/.

## Appendix A

**Table A1:** Socio-demographic variables. Results are shown in absolute and relative (%) frequencies.

Personal Characteristics	Level	*n* (%)
Sex	Male	152 (23.4)
	Female	495 (76.3)
	Other	2 (0.3)
Nationality	Portuguese	616 (94.9)
	Brazilian	30 (4.6)
	Chinese	1 (0.15)
	Colombian	1 (0.15)
	French	1 (0.15)
Religion	No religion	302 (46.5)
	Christian	278 (42.7)
	Buddhism	2 (0.3)
	Asatru	1 (0.2)
	Spiritualist	5 (0.8)
	Evangelist	5 (0.8)
	Jehovah’s Witness	1 (0.2)
	Protestant	1 (0.2)
	Any other religion	49 (7.5)
	Prefer not to say	5 (0.8)
Highest qualification	Primary	2 (0.3)
	Basic	21 (3.2)
	Secondary	179 (27.6)
	Bachelor	258 (39.8)
	Master	172 (26.5)
	Doctorate	11 (1.7)
	Other technical, professional, or higher qualification	6 (0.9)
Socioeconomic status	Low	192 (29.5)
	Medium	380 (58.6)
	High	77 (11.9)
Employment status	Employed	212 (32.7)
	Student	288 (44.4)
	Working student	52 (8.0)
	Unemployed	58 (8.9)
	Retired	18 (2.8)
	Other	21 (3.2)

## Appendix B

**Table A2:** Principal component loadings, following varimax rotation for items relating to attitudes and beliefs about COVID-19.

	Perceived Threat of COVID-19	Trust in Management of COVID-19	Impact of COVID-19
I am worried about catching coronavirus	−0.377		
I believe that coronavirus would be a mild illness for me	0.514		
I believe I am immune to coronavirus	0.493		
I trust the NHS to manage the coronavirus pandemic in Portugal		0.693	
I trust the government to manage the coronavirus pandemic in Portugal		0.650	
The coronavirus pandemic has had a big impact on my life			0.91

## Appendix C

**Table A3:** Principal component loadings, following varimax rotation for items relating to attitudes and beliefs about the COVID-19 vaccine.

	General COVID-19 Vaccination Beliefs and Attitudes	Others’ Intention	Perceived Knowledge Sufficiency	Return to Normal Life
A coronavirus vaccination should be mandatory for everyone who is able to have it	0.550			
Without a coronavirus vaccine, I am likely to catch coronavirus	0.498			
If I get a coronavirus vaccination, I will be protected against coronavirus	0.658			
If I don’t get a coronavirus vaccination and end up getting coronavirus, I would regret not getting the vaccination	0.647			
It would be very easy for me to have a coronavirus vaccination	0.753			
A coronavirus vaccination could give me coronavirus	−0.492			
I would be worried about experiencing side effects from a coronavirus vaccination	−0.557			
I might regret getting a coronavirus vaccination if I later experienced side effects from the vaccination	−0.583			
A coronavirus vaccination will be too new for me to be confident about getting vaccinated	−0.793			
Most people will get a coronavirus vaccination	-	0.669		
Other people like me will get a coronavirus vaccination	-	0.572		
If I were vaccinated, I think I would not need to follow social distancing and other restrictions for coronavirus				0.574
I know enough about the coronavirus illness to make an informed decision about whether or not to get vaccinated			0.585	
I know enough about the coronavirus vaccine to make an informed decision about whether or not to get vaccinated			0.569	
My family would approve of my having a coronavirus vaccination	0.665			
My friends would approve of my having a coronavirus vaccination	0.646			
If a coronavirus vaccination were recommended by the government, I would get vaccinated	0.621			
If a coronavirus vaccination were recommended by a health care professional (e.g., doctor), I would get vaccinated	0.788			
A coronavirus vaccine will allow us to get back to normal	0.663			
There would be no point in having the coronavirus vaccination unless I could go back to my normal life				0.604

## Appendix F

**Table A4:** Confusion matrix for the predicted vs. real values in the testing set.

Predicted			
	Low	Moderate	High
Low	11	3	0
Moderate	0	24	10
High	0	7	75

**Table A5:** Evaluation metrics for each class of the classification problem.

Class	Precision	Recall	F1 Score
Low	100%	79%	88%
Moderate	71%	71%	71%
High	88%	91%	90%
